# Changing trends of patient characteristics and treatment pathways during the COVID-19 pandemic: A cross-sectional analysis of 72,459 inpatient cases from the German Helios database

**DOI:** 10.3389/fpubh.2022.1028062

**Published:** 2022-11-07

**Authors:** Sebastian König, Sven Hohenstein, Vincent Pellissier, Johannes Leiner, Gerhard Hindricks, Irit Nachtigall, Ralf Kuhlen, Andreas Bollmann

**Affiliations:** ^1^Department of Electrophysiology, Heart Center Leipzig at University of Leipzig, Leipzig, Germany; ^2^Real World Evidence and Health Technology Assessment, Helios Health Institute, Berlin, Germany; ^3^Department of Preventive Medicine and Hygiene, Helios Hospital Bad Saarow, Bad Saarow, Germany; ^4^Department of Anaesthesiology and Operative Intensive Care Medicine, Charité-Universitätsmedizin Berlin, Berlin, Germany; ^5^Helios Health, Berlin, Germany

**Keywords:** COVID-19, patient characteristics, trend analysis, in-hospital mortality, administrative data

## Abstract

**Background:**

This study compared patient profiles and clinical courses of SARS-CoV-2 infected inpatients over different pandemic periods.

**Methods:**

In a retrospective cross-sectional analysis, we examined administrative data of German Helios hospitals using ICD-10-codes at discharge. Inpatient cases with SARS-CoV-2 infection admitted between 03/04/2020 and 07/19/2022 were included irrespective of the reason for hospitalization. All endpoints were timely assigned to admission date for trend analysis. The first pandemic wave was defined by change points in time-series of incident daily infections and compared with different later pandemic phases according to virus type predominance.

**Results:**

We included 72,459 inpatient cases. Patients hospitalized during the first pandemic wave (03/04/2020–05/05/2020; *n* = 1,803) were older (68.5 ± 17.2 vs. 64.4 ± 22.6 years, *p* < 0.01) and severe acute respiratory infections were more prevalent (85.2 vs. 53.3%, *p* < 0.01). No differences were observed with respect to distribution of sex, but comorbidity burden was higher in the first pandemic wave. The risk of receiving intensive care therapy was reduced in all later pandemic phases as was in-hospital mortality when compared to the first pandemic wave. Trend analysis revealed declines of mean age and Elixhauser comorbidity index over time as well as a decline of the utilization of intensive care therapy, mechanical ventilation and in-hospital mortality.

**Conclusion:**

Characteristics and outcomes of inpatients with SARS-CoV-2 infection changed throughout the observational period. An ongoing evaluation of trends and care pathways will allow for the assessment of future demands.

## Introduction

The ongoing coronavirus disease 2019 (COVID-19) pandemic still affects global health care according to high case numbers of new infections (1). There have been several studies reporting on patient characteristics in its early phase between March and May 2020 (2–6). However, only insufficient data is available with regard to changes of patient profiles, treatment pathways and outcome differences when comparing the first pandemic wave with periods thereafter. Studies focusing on such trend analyzes either described specific subgroups like those who required intensive care treatment, were of small sample size or did not included information on cases hospitalized beyond early 2021 and therefore not adequately covered the later pandemic phases (7–14). Since a deeper understanding of the developments with regard to COVID-19-related health care is critical in anticipation of future requirements, aim of this study was to describe baseline characteristics as well as temporal trends of patients' treatments and outcomes throughout the COVID-19 pandemic in a nationwide German inpatient database.

## Methods

### Data collection

In this retrospective, cross-sectional study, we analyzed administrative data of 87 Helios hospitals in Germany. All completed inpatient cases with a laboratory confirmed active severe acute respiratory syndrome coronavirus type 2 (SARS-CoV-2) infection and an admission date between March 4th 2020 and July 19th 2022 were included irrespective of the reason for hospitalization. Relevant comorbidities were assessed based on the encoded primary and secondary diagnoses at hospital discharge according to the International Statistical Classification of Diseases and Related Health Problems [ICD-10-GM (German Modification)]. SARS-CoV-2 infection (U07.1) and severe acute respiratory infections (SARI; J09-J22) were identified *via* ICD-codes. Comorbidities were structured using a modified ICD-10-based Elixhauser comorbidity index using the R comorbidity package with detailed information on used ICD-10 codes being provided in the Supplementary Table 1 (15–17). Operations and Procedures-codes [OPS (German adaptation of the International Classification of the Procedures in Medicine of the World Health Organization, version 2017)] and administrative data-derived information regarding in-hospital treatments were used to specify outcomes such as intensive care therapy (8-980/8-98f/duration of intensive care-stay >0 days), invasive mechanical ventilation (8-70x/8-71x/duration of ventilation >0 days), or treatment with an extra-corporal membrane oxygenation (ECMO; 8-852.0/8-852.3/8-852.6). Hospital discharge type, length of stay, length of intensive care treatment (including duration of mechanical ventilation) and healthcare associated costs were extracted from administrative data. For in-hospital mortality analysis, cases discharged as hospital transfers or with unspecified discharge type were excluded. All endpoints were timely assigned to the case admission date for trend analysis. There was no missing data. Patients' data were stored in a double-pseudonymized form and data use was approved by the local ethics committee of the University of Leipzig and the Helios Kliniken GmbH data protection authority. Considering the retrospective analysis of double-pseudonymized administrative clinical routine data, individual informed consent was not obtained. The study follows the STROBE guidelines for cross-sectional analyzes. Governmental and public health interventions throughout the pandemic course were assessed and displayed (Supplementary Figure 1) by using data from the Robert-Koch-Institute as well as the German Ministry of Health.

### Statistical analysis

**Figure 1:**
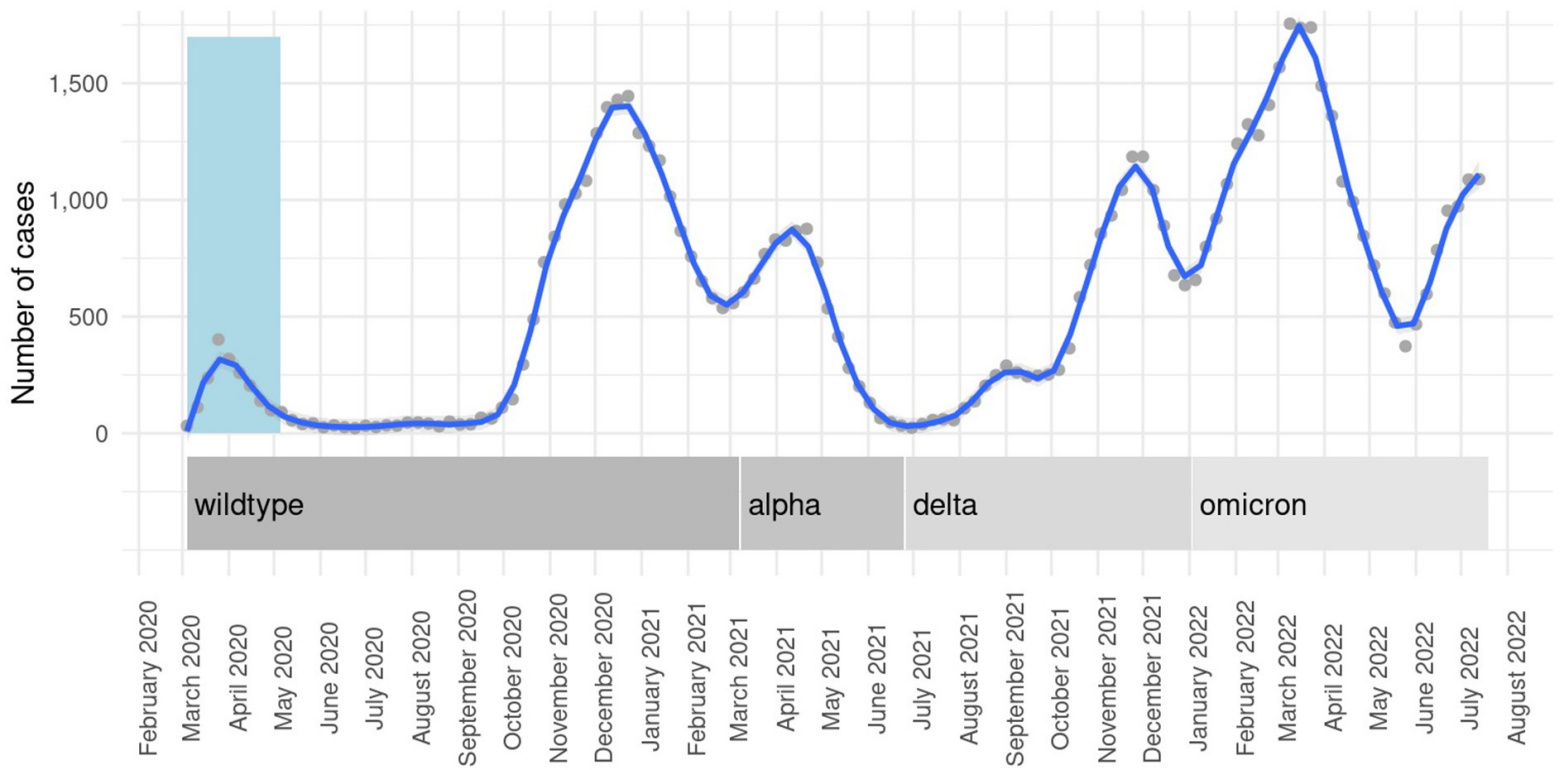
Weekly SARS-CoV-2 admissions and corresponding variant predominance throughout the pandemic. Locally estimated scatterplot smoothing curves with 95% CIs for weekly case numbers as a function of admission week. Light-blue column represents the first pandemic wave.

Administrative data were extracted from QlikView (QlikTech, Radnor, Pennsylvania, USA). Inferential statistics were based on generalized linear mixed models (GLMM) specifying hospitals as random factors with and without adjustment for age, sex and Elixhauser comorbidity index (18). We employed logistic GLMMs with logit link function for binary data. Effects were estimated with the lme4 package (version 1.1-26) in the R environment for statistical computing (version 4.0.2) (19, 20). Varying intercepts for the random factor were specified in all models. We report odds ratios [OR, calculated by exponentiation of the regression coefficients (RC)] together with 95% confidence intervals (CI) and *p*-values. Linear mixed models were used for the analysis of numerical variables, and RCs together with CIs were reported. A log-transformation of positively skewed variables (durations of treatment) was done in order to approximate normal distributions. Different pandemic phases were defined based on the time-series of daily registered SARS-CoV-2 infections including predominant virus variants since January 2020 as reported by the Robert-Koch-Institute. Within the phase of wildtype virus predominance, the first pandemic wave has been distinguished from the periods thereafter by change points in time-series data applying a segmented linear regression (Figure 1). Including an extension of the wave's duration to encompass full weeks, an interval from March 4th to May 5th 2020 resulted for the first wave's definition. Later pandemic periods corresponding to the dominance of different virus variants were: wildtype after first wave: May 6th 2020 to March 7th 2021; alpha dominance: March 8th 2021 to June 25th 2021; Delta dominance: June 26th 2021 to January 2nd 2022; Omicron dominance: January 3rd 2022 to July 19th 2022. For the multivariable analyzes, numerical variables were centered on their mean, and sex was specified as 0.5 vs. −0.5. Pandemic periods entered the analysis as comparisons with the reference level (first wave).

For the analysis of temporal linear trends, we scaled the day indices (predictor variable) to an interval from zero (1st day) to one (last day). Numerical dependent variables were scaled to zero mean and unit variance. All cases entered these analyzes without aggregation. Trend analyzes with corresponding graphs were created using the ggplot2 package within R (21).

The sliding analyses represent repeated comparisons with the first wave period (baseline) adjusted for age, sex and Elixhauser comorbidity index (age and Elixhauser comorbidity index were included as continuous variables for all model adjustments). Starting with this period, the 9-week interval was repeatedly shifted by 1 week at a time (test period). In each analysis, we calculated means for the dependent variable in the test period and ORs with corresponding CIs. Although all periods encompassed the same number of days, the number of observations was subject to variance. As part of a sensitivity analysis, sliding 9-week ORs of in-hospital mortality were calculated stratifying for the utilization of intensive care therapy. Trends as a function of time were used to illustrate results of the sliding trend analysis representing partial effects of the days on the dependent variable, which were calculated by removing random effects (of the hospitals) and effects of the covariates from this variable. Trend analysis were presented graphically *via* locally estimated scatterplot smoothing curves (degree of smoothing α = 0.08) together with 95% CIs. For all tests we applied a two-tailed 5% error criterion for significance.

## Results

### Baseline characteristics

Of 87 hospitals, 86 centers contributed in total 72,459 inpatient cases with confirmed SARS-CoV-2 infection to the database. Of them, 14,353 (1,803 during the first wave), 28,325 and 29,781 cases were admitted in 2020, 2021, and 2022, respectively. Mean age of the overall cohort was 64.5 ± 22.5 years and 48.8% (*n* = 35,333) were women. Comorbidities were frequent and SARI was encoded in more than half of cases (54.1%, *n* = 39,185). When comparing baseline characteristics of the first pandemic wave and the cumulative period thereafter (Table 1), a higher mean age (68.5 ± 17.2 vs. 64.4 ± 22.6 years, *p* < 0.01) and a higher proportion of cases with SARI (85.2 vs. 53.3%, *p* < 0.01) were observed in the first period. There were no differences with respect to sex distribution, but cases hospitalized within the first pandemic wave had a higher Elixhauser comorbidity index (11.0 ± 11.6 vs. 9.8 ± 11.2, *p* < 0.01). A more detailed comparison of baseline characteristics including the differentiation of comorbidity groups is provided in the Supplementary Table 2. Subdividing pandemic phases after the first wave according to the predominant virus variant, hospitalized SARS-CoV-2 cases were younger and suffered from less comorbidities according to a lower Elixhauser comorbidity index in alpha, delta and omicron dominance periods, but not in the interval with wildtype predominance after the first wave. The proportion of cases fulfilling SARI criteria was significantly lower in all later pandemic phases when compared to the first wave. More detailed results of this comparison of distinct pandemic periods are provided in the Supplementary Table 3.

**Table 1:** Baseline characteristics overall and comparing the first pandemic wave with the period thereafter.

**Proportion (*n*)**			
**Variable**	**First wave**	**After first wave**	***P*** **-value**
**Age**			
*mean (SD)* [years]	68.5 ± 17.2	64.4 ± 22.6	<0.01
≤59 years	28.3% (510)	33.1% (23,355)	<0.01
60–69 years	16.1% (290)	15.5% (10,943)	0.14
70–79 years	23.1% (417)	19.5% (13,772)	<0.01
≥80 years	32.5% (586)	32.0% (22,586)	0.96
**Sex**			
Male	51.9% (936)	51.2% (36,190)	
Female	48.1% (867)	48.8% (34,466)	0.35
**SARI**			
No	14.8% (267)	46.7% (33,007)	
Yes	85.2% (1,536)	53.3% (37,649)	<0.01
**Elixhauser comorbidity index**			
*mean (SD)*	11.0 ± 11.6	9.8 ± 11.2	<0.01
<0	13.3% (240)	13.8% (9,724)	0.78
0	16.7% (301)	19.9% (14,066)	<0.01
1–4	5.5% (99)	5.5% (3,899)	0.87
≥5	64.5% (1,163)	60.8% (42,967)	<0.01

Analysis is based on linear (continuous variables) or logistic (categorical variables) mixed models.

SARI, Severe acute respiratory infections.

### Treatments and outcomes

Throughout all cases, mean length of stay was 11.2 ± 14.4 days and 22.3% (*n* = 16,144) received an intensive care treatment (mean length of stay at intensive care facility 9.0 ± 12.5 days) with 13.2% (*n* = 9,590) and 0.5% (*n* = 378) of cases requiring invasive mechanical ventilation and ECMO therapy. Mean duration of mechanical ventilation was 241.6 ± 296.4 h. In-hospital mortality was 14.2% overall with higher mortality rates within subgroups of specific intensive care therapies (intensive care treatment: 33.5%, mechanical ventilation: 52.4%, ECMO: 81.8%). Comparing in-hospital treatments with the first pandemic wave, cases admitted thereafter were less likely to receive intensive care treatment (33.1 vs. 22.0%, *p* < 0.01), mechanical ventilation (20.1 vs. 13.1%, *p* < 0.01) as well as ECMO therapy (0.7 vs. 0.5%, *p* < 0.01). The reduced utilization of intensive care treatment and mechanical ventilation when compared to the first wave was consistent throughout distinct later pandemic phases except from the rate of invasive ventilation during alpha predominance (Supplementary Table 4). ECMO use remained stable during alpha and delta dominance intervals, but was reduced when omicron was the dominant virus variant. Mean length of intensive care treatment (12.1 ± 15.0 vs. 8.9 ± 12.4 days, *p* < 0.01) and the duration of mechanical ventilation (321.5 ± 344.1 vs. 238.0 ± 293.5, *p* < 0.01) were longer during the first wave as was the overall length of stay (14.6 ± 26.4 vs. 11.1 ± 13.9 days, *p* < 0.01). In-hospital mortality was reduced in the aggregated “after first wave” period (22.4 vs. 14.1%, *p* < 0.01) and throughout all distinct later pandemic periods with a marked decrease during the omicron predominance (Supplementary Table 4). A comparison of inpatient treatment durations for distinct pandemic phases according to virus predominance periods is given in the Supplementary Table 5. In multivariable analysis, later pandemic periods were shown as independent predictors for a less frequent occurrence of SARI cases, decreased utilizations of intensive care therapy and a lower in-hospital fatality rate (Table 2). The risk of receiving mechanical ventilation was increased during the alpha predominance period when compared with the first pandemic wave whereas a markedly reduced risk was found for the period in which omicron was the dominant virus variant (Table 2).

**Table 2:** Multivariable analysis for occurrence of SARI and in-hospital outcomes.

**Variable**	**SARI**		**Intensive care**		**Mechanical ventilation**		**In-hospital mortality** ^*^	
	**OR (95% CI)**	***P*-value**	**OR (95% CI)**	***P*-value**	**OR (95% CI)**	***P*-value**	**OR (95% CI)**	***P*-value**
Age	1.46 (1.43–1.49)	<0.01	1.04 (1.01–1.06)	<0.01	0.94 (0.92–0.97)	<0.01	1.06 (1.05–1.06)	<0.01
Male sex	1.58 (1.53–1.63)	<0.01	1.56 (1.50–1.62)	<0.01	1.76 (1.68–1.84)	<0.01	1.60 (1.52–1.68)	<0.01
Elixhauser comorbidity index	1.29 (1.26–1.31)	<0.01	1.78 (1.75–1.82)	<0.01	1.85 (1.81–1.89)	<0.01	1.06 (1.05–1.06)	<0.01
Wildtype after first wave	0.44 (0.38–0.50)	<0.01	0.68 (0.60–0.76)	<0.01	0.71 (0.62–0.81)	<0.01	0.76 (0.66–0.87)	<0.01
Alpha	0.73 (0.63–0.84)	<0.01	0.86 (0.76–0.96)	0.010	1.17 (1.02–1.35)	0.021	0.74 (0.63–0.86)	<0.01
Delta	0.44 (0.38–0.50)	<0.01	0.73 (0.65–0.82)	<0.01	0.93 (0.81–1.06)	0.288	0.78 (0.67–0.90)	<0.01
Omicron	0.50 (0.45–0.54)	<0.01	0.83 (0.77–0.89)	<0.01	0.47 (0.43–0.51)	<0.01	0.62 (0.56–0.68)	<0.01

Each virus predominance period is compared with the first pandemic wave.

* Based on 67,913 cases after the exclusion of cases with hospital discharge type of hospital transfer or unspecified reason.

CI, Confidence interval; OR, Odds ratio; SARI, Severe acute respiratory infections.

### Trend analysis

**Figure 2:**
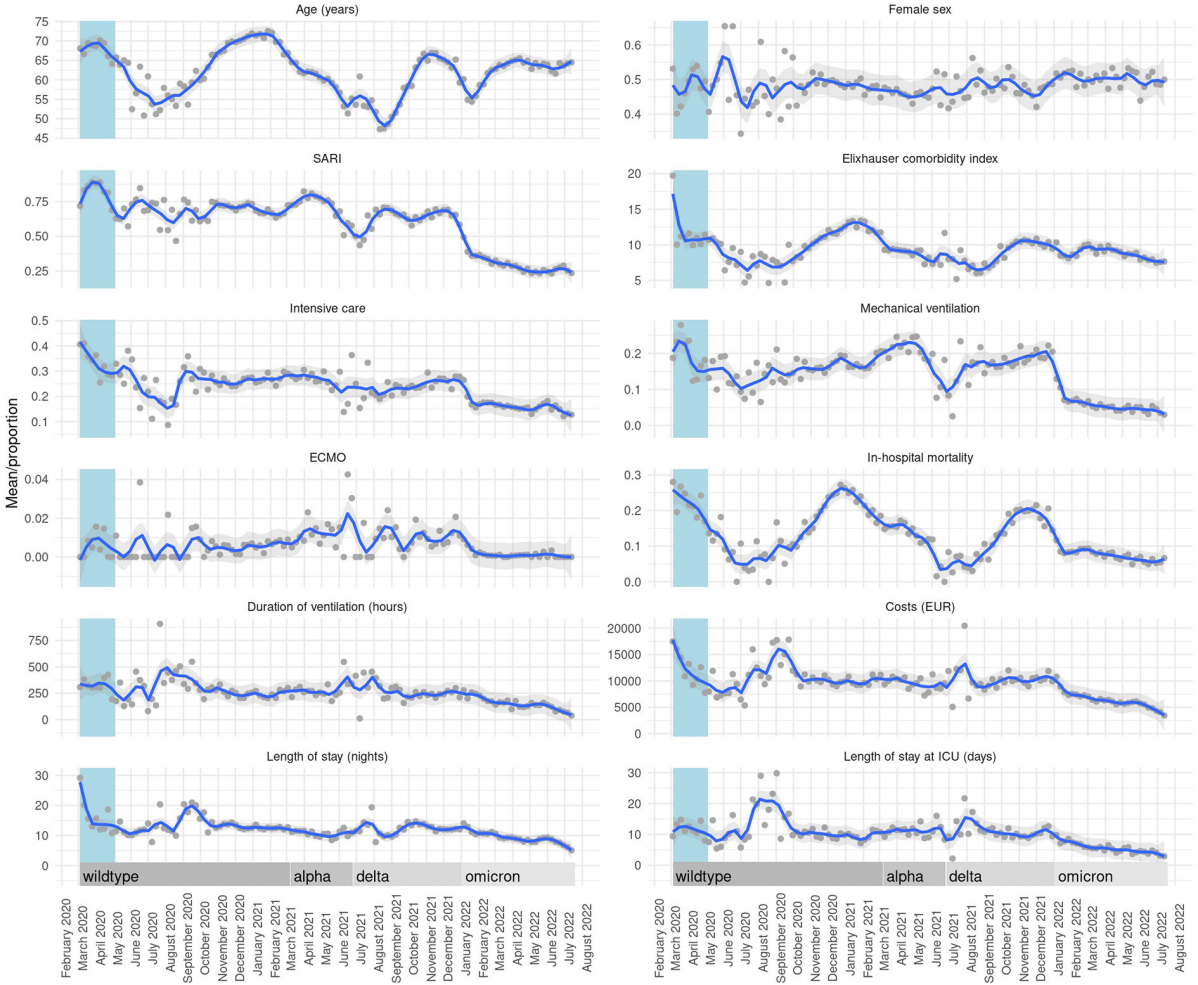
Temporal trends presented as weekly means/proportions per variable. Locally estimated scatterplot smoothing curves with 95% CIs for weekly means/proportions as a function of admission week.

**Figure 3:**
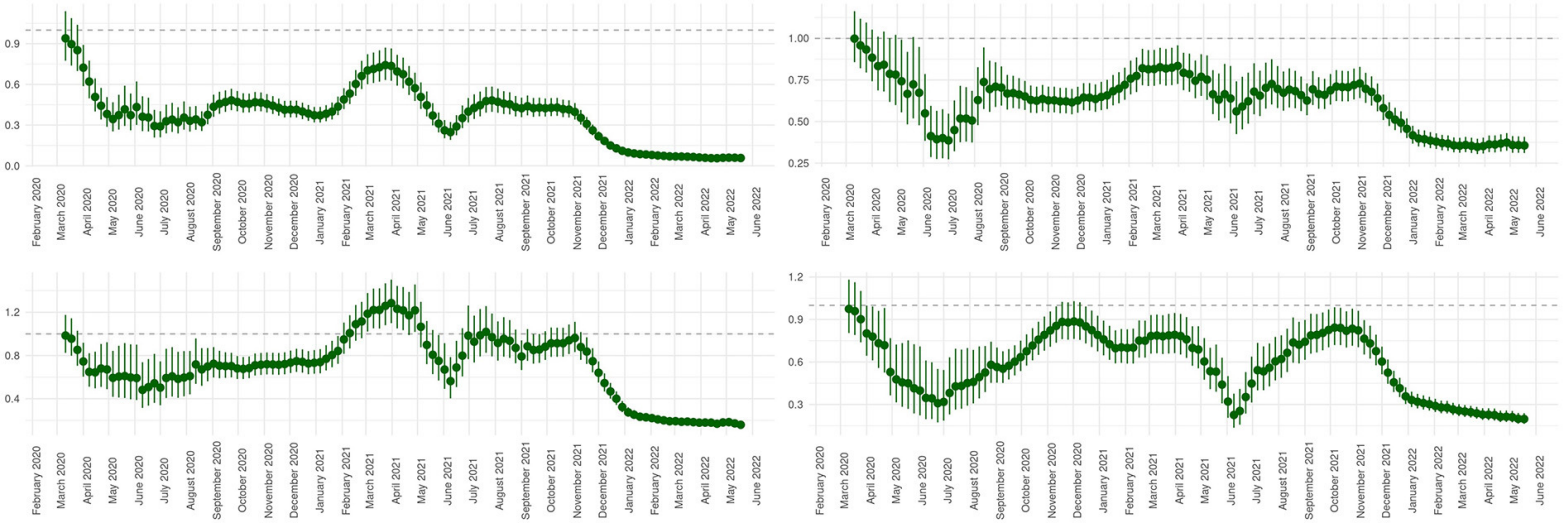
Sliding comparison of 9-week periods with the first pandemic wave investigating occurrence of SARI, intensive care therapy, mechanical ventilation, and in-hospital mortality. The presentation of 9-week periods (sliding ORs) that are temporarily assigned to the beginning of the interval lead to a time shift of the corresponding trends when being compared to Figure 2. For the same reason the graphs end before the end of the study period. All shown analyzes are adjusted for age, gender, and Elixhauser comorbidity index. **(Upper left panel)** Sliding comparison of the proportion of encoded SARI. **(Upper right panel)** Sliding comparison of the proportion of patients receiving intensive care therapy. **(Lower left panel)** Sliding comparison of the proportion of patients receiving mechanical ventilation. **(Lower right panel)** Sliding comparison of in-hospital mortality rates.

Calculating linear trends over time, we observed a reduction of mean and Elixhauser comorbidity index. We observed a decreasing occurrence of SARI as well as a decreasing utilization of intensive care therapy, mechanical ventilation and use of ECMO therapy over time. In-hospital mortality declined throughout the pandemic course. Length of stay as well as the duration of intensive care treatment and mechanical ventilation shortened over time. This was accompanied by a trend of decreasing healthcare costs (RC −0.60, 95% CI −0.63 to −0.58, *p* < 0.01). Detailed results of the temporal trend analyzes are provided in Table 3 and visualized as weekly means or proportions per variable in Figure 2. Sliding 9-week ORs were calculated in a multivariable model adjusting for age, sex and the Elixhauser comorbidity index for SARI, intensive care treatment, mechanical ventilation and in-hospital mortality. Results are presented in Figure 3. The overall trend within sliding 9-week ORs of in-hospital mortality was consistent in patients with and without intensive care therapy (Supplementary Figure 2).

**Table 3:** Linear trends over time.

**Variable**	**Odds ratio (95% CI)**	**Regression coefficient (95% CI)**	***P*-value**
Age [years]	/	−0.35 (−0.37 to −0.32)	<0.01
Female sex	1.09 (1.03–1.16)	/	0.003
SARI	0.05 (0.04–0.05)	/	<0.01
Elixhauser comorbidity index	/	−0.31 (−0.34 to −0.29)	<0.01
Intensive care	0.36 (0.34–0.39)	/	<0.01
Mechanical ventilation	0.20 (0.18–0.22)	/	<0.01
ECMO	0.23 (0.15–0.34)	/	<0.01
LoS [days]	/	−0.64 (−0.67 to −0.61)	<0.01
LoS intensive care unit [days]^*^	/	−0.72 (−0.78 to −0.66)	<0.01
Duration of mechanical ventilation [hours]^†^	/	−0.63 (−0.72 to −0.54)	<0.01
In-hospital mortality^‡^	0.20 (0.18–0.21)	/	<0.01

* Based on 12,440 cases (19.2%). We excluded cases with length of stay at ICU = 0.

† Based on 5,620 cases (8.7%). We excluded cases with duration of ventilation = 0.

‡ Based on 64,571 cases (99.7%). We excluded cases with discharge due to hospital transfer or unspecified reason.

CI, Confidence interval; ECMO, Extra-corporal membrane oxygenation; LoS, Length of stay; SARI, Severe acute respiratory infections.

## Discussion

With this retrospective, cross-sectional analysis, we provide insights into the temporal evolution of baseline characteristics, treatment courses and outcomes of patients with laboratory proven SARS-CoV-2 infection who were treated in one of 86 Helios hospitals across Germany between March 4th 2020 and July 19th 2022. Comparing different periods, inpatients of the first pandemic wave were older, had more comorbidities and required more often an intensive care therapy than the patients being hospitalized thereafter. Trend analysis revealed a decrease in in-hospital mortality over time. Interestingly, there were seasonal variations to be observed with regard to trend curves of mean age of hospitalized patients, mean Elixhauser comorbidity index and in-hospital mortality rate whereas no such relationship was obvious for other investigated variables including the use of intensive care therapy or mechanical ventilation.

Baseline characteristics of inpatient cases within the first pandemic wave period varied relevantly between published cohorts with our patients' age and comorbidity distribution being within the reported range (3, 6). Of note, compared to two previously presented German cohorts with one of them also investigating patients from selected Helios hospitals, mean age of cases included into our analysis was lower but other baseline characteristics were comparable (22, 23). Intensive care treatment as well as mechanical ventilation were required in a higher proportion of cases from our cohort when compared to multicentric analyzes from the United States or the United Kingdom. Despite this increased use of intensive care, in-hospital mortality rate was lower than those presented in the studies of Richardson et al. and Docherty et al., respectively (3, 6). Other groups reported in-hospital fatal outcomes ranging from 10% to more than 30% of patients during the first pandemic wave, but corresponding groups were of smaller size and not comparable with respect to patients profiles (4, 5, 24, 25). The identification of age, male sex and comorbidity burden as predictors for intensive care treatment and in-hospital death is in line with previous findings (3). The reduced OR for mechanical ventilation in older patients is probably to be interpreted more in the sense of fa conscious medical consideration with regard to the implementation of such an invasive therapy than as an actual reduced risk.

Previously published data on changes of inpatient characteristics and treatment paths comparing the first pandemic wave with later pandemic phases is currently limited to data of early 2021 and therefore not covers the evolution of the pandemic thereafter. Later reports focused on a comparison of single virus variants like delta and omicron, but did not investigate changing treatment patterns over time. Looking first at studies reporting outcome changes until mid-2020, patients admitted after the first wave were characterized to be younger and have a favorable in-hospital death probability (26). Inconsistent observations were made with respect to comorbidity burden, which was related to differing cohort definitions (e.g., intensive care patients only vs. all hospitalized patients) (13, 27). Reports extending their period of interest to the upstroke of the second pandemic wave showed similar results. Patients being hospitalized after the first wave were younger, had less comorbidities and a milder disease presentation leading to a reduced short-term mortality even after the adjustment for covariates (7, 12, 28). However, the evolution of mortality rates was not uniform in between different investigations. Comparable to our findings, Lefrancq et al. found a *U*-shaped curve of the overall in-hospital mortality rate until the end of 2020 (11). Conversely, a steady decrease of mortality rates was reported in an US COVID-19 registry from March to November 2020 (12). Taking into account the later rise of case numbers as a marker of a shifted beginning of the second wave in the USA when compared to France and Germany, those results do not necessarily stay in contrast to our observations. When examining proportions of patients receiving an intensive care treatment, the French group also reported a decrease toward summer 2020 with a re-increase at the beginning of the second wave. It is noteworthy that the absolute percentages are remarkably lower compared to our results despite a rather higher total mortality (11). The direct comparison is, however, hindered by the lacking information with regard to comorbidity burden and the overall low probability of an intensive care therapy in patients aged 70 years or older. Interestingly, the authors provided correlation analysis accounting for occupancy of the intensive care unit showing no association with overall mortality (11). Karagiannidis et al. reported data on COVID-19 related patient care until the end of 2020 for Germany. The authors found a relative reduction with regard to the proportion of patients receiving an intensive care treatment as well as mechanical ventilation (10). Although a comparable trend was depicted by our analyzes, the percentages presented were strikingly low compared to our analysis. One has to consider that all analyzes that only include 2020 data do not take into account the climax of the second wave and the development of the pandemic thereafter, which is likely to influence results. Aside of a possible bias due to the selection of hospitals contributing patient cases to our database, a different definition of intensive care treatment could be an influencing factor to those discrepancies. In another rather smaller study, percentages of intensive care requirement were comparable to those of our cohort (8). Focusing on studies that extended their observational period to include the third pandemic wave in early 2021, comparable observations of a decrease of the proportion of patients receiving mechanical ventilation and lower in-hospital mortality were made (29–31). Moreover, our findings of a decreasing age of hospitalized patients as well as a shortened length-of-stay were confirmed (30, 31). Of note, in the report of Xia et al., which partly covers the pandemic phase with alpha virus variant dominance, no specific risk for the utilization of mechanical ventilation is provided. Since other studies only insufficiently covered this particular pandemic period, a comparison of findings to the increased risk for receiving mechanical ventilation as found in our multivariate analysis is hindered. Recently, there have been reports comparing inpatient outcomes of cases with SARS-CoV-2 infection from delta and omicron variants. In has been confirmed that the risk of severe disease courses as well as in-hospital death were reduced during omicron variant predominance, which is in line with our findings (32–34). However, data investigating treatment patterns and outcomes throughout the whole pandemic is lacking.

There are a number of possible reasons to explain the diverging characteristics between first waves' patients and those being hospitalized with SARS-CoV-2 infection thereafter. The reduced mean age of inpatient cases admitted after the first wave might be a consequence of behavioral, structural and social adjustments that were introduced to particularly protect older and more vulnerable population groups. There were several governmental as well as public health interventions that were repetitively adapted over time. A selection of relevant public health measures is depicted in the Supplementary Figure 1. The decreasing trend of age and comorbidity burden in hospitalized patients throughout the pandemic could also be an effect of the evolving vaccination program in Germany with higher rates of fully vaccinated individuals in older person groups. The observations of a lower mortality throughout the pandemic course compared to the early phase are potentially related to growing experiences in the treatment of COVID-19 patients including an increased use of glucocorticoids and other supporting therapies (9, 14, 35, 36). For example, nasal high-flow therapy and non-invasive assistant ventilation were utilized more often after the first wave period (14, 37). Therefore, the lower proportion of patients receiving invasive ventilation may both be a consequence of an actual reduced rate of severe cases and a conscious decision to delay the use of this treatment modality. It has to be pointed out that a re-increase of in-hospital mortality to the initial first waves' level was observed during the peak of the second wave. The above made considerations therefore reach their limits when the health care system is under heavy load of COVID-19 patient numbers. However, in waves thereafter, in-hospital mortality peaked at a lower level which, in addition to the aspects already mentioned, might also be related to changing predominant variants of the virus. Upon others, the latter assumption is supported by reports that indicate a lower pathogenicity of the omicron variant when compared to previous virus variants (38–40).

Of course, our study only reflects data from Germany. However, comparable observations of the examined endpoints across several countries in Europe and North America during the early pandemic phases suggest partial transferability of our findings. Nevertheless, a further scientific evaluation and confirmation of our results is required.

## Limitations

This study is based on administrative data that was not stored for research interests but for remuneration reasons, which potentially could affect the encoded information and harbors the risk of bias in the evaluation of various clinical endpoints (41). Quality of the results depends to a large extent on the correct encoding of procedures and diagnoses at hospital discharge (17). This is particularly true for the encoding of SARS-CoV-2-infection, as the specific ICD-code has been introduced at April 1st 2020 and was retrospectively encoded thereafter for all previous cases. However, regarding the discharge diagnoses and the adequacy of hospitalization as well as encoding, there is a continuous evaluation by reimbursement companies/health insurances which supports the assumption of overall valid information, which also accounts for the supplemental information regarding the SARS-CoV-2 status as it is relevant for reimbursement. Due to the type of data, no specific causes of death could be determined and additional supporting information regarding patients' specific medical history, imaging, laboratory results, medication and treatment-related data was not available. Moreover, due to the data structure, no linking of patient data between different hospitals was possible. Therefore, hospital transfers both at admission and discharge may influenced results. However, repeating the trend analyzes after the exclusion of all patients admitted or discharged as a hospital transfer, similar results were found.

## Conclusion

This is the first study comparing patient characteristics and outcomes of the first wave of the ongoing COVID-19 pandemic with the periods thereafter up to mid-2022. We observed trends toward a reduction of mean age and the presence of relevant comorbidities as well as in-hospital mortality in inpatients with proven SARS-CoV-2 infection. An ongoing evaluation is essential in order to be able to assess future demands on the health care system.

## Data availability statement

The raw datasets presented in this article are not readily available for data protection reasons. Requests to access the datasets should be directed to the corresponding author of this article.

## Ethics statement

The studies involving human participants were reviewed and approved by Ethik-Kommission der Medizinischen Fakultät der Universität Leipzig. Written informed consent for participation was not required for this study in accordance with the national legislation and the institutional requirements.

## Author contributions

SK, RK, and AB were largely responsible for the conception, planning, and the conduct of the investigation. SH and VP were responsible for data acquisition, statistical planning, and formal analysis. SK was responsible for writing the first draft of the manuscript. SK, JL, GH, IN, RK, and AB were responsible for drafting and revising the work. All authors approved the final version for submission and subsequent publication.

## Conflict of interest

The authors declare that the research was conducted in the absence of any commercial or financial relationships that could be construed as a potential conflict of interest.

## Publisher's note

All claims expressed in this article are solely those of the authors and do not necessarily represent those of their affiliated organizations, or those of the publisher, the editors and the reviewers. Any product that may be evaluated in this article, or claim that may be made by its manufacturer, is not guaranteed or endorsed by the publisher.

## Abbreviations

AIC: Akaike's Information Criterion
CI: Confidence interval
COVID-19: Coronavirus disease 2019
ECMO: Extra-corporal membrane oxygenation
GLMM: Generalized linear mixed models
ICD-10: International Statistical Classification of Diseases and Related Health Problems, 10th revision
OPS: Operations and Procedures
OR: Odds ratio
SARI: Severe acute respiratory infections
SARS-CoV-2: Severe acute respiratory syndrome coronavirus type 2.
