# Supplementary material for: Changing trends of patient characteristics and treatment pathways during the COVID-19 pandemic: A cross-sectional analysis of 72,459 inpatient cases from the German Helios database
Source: Front Public Health. 2022 Nov 7;10:1028062. doi: 10.3389/fpubh.2022.1028062 (PMC9678052; doi:10.3389/fpubh.2022.1028062)
Supplement: Supplementary file 1 [file Data_Sheet_1.DOCX]

**Supplemental Material**

Supplemental Table 1: ICD-codes used to calculate Elixhauser Comorbidity Score (weighting according to AHRQ algorithm)

Supplemental Table 2: Detailed baseline characteristics overall and comparing the first pandemic wave with the period thereafter

Supplemental Table 3: Comparison of baseline characteristics dividing five pandemic periods based on incident new SARS-CoV-2 cases

Supplemental Table 4: Comparison of in-hospital outcomes dividing five pandemic periods based on incident new SARS-CoV-2 cases

Supplemental Table 5: Comparison of inpatient pathways and costs dividing four pandemic periods based on incident new SARS-CoV-2 cases

Supplemental Figure 1: Graphical overview on pandemic development over time in Germany including public health interventions

Supplemental Figure 2: Sliding comparison of in-hospital mortality in 9-week periods with the first pandemic wave stratified for intensive care therapy

Legend: Sliding comparison of in-hospital mortality rates (adjusted for age, gender and Elixhauser comorbidity index) for patients receiving intensive care therapy (left panel) and those who did not (right panel)

**Supplemental Table 1: ICD-codes used to calculate Elixhauser Comorbidity Score**

| ICD*-codes used to calculate Elixhauser comorbidity score | | |
| --- | --- | --- |
| *Item* | *Weight* | *ICD*-10-GM-code* |
| AIDS / HIV | 0 | B20, B21, B22, B23, B24 |
| Alcohol Abuse | -1 | F10, E52, G62.1, I42.6, K29.2, K70.0, K70.3, K70.9, T51, Z50.2, Z71.4, Z72.1 |
| Blood Loss Anemia | -3 | D50.0 |
| Cardiac Arrhythmias | 0 | I44.1, I44.2, I44.3, I45.6, I47, I48, I49, R00.0, R00.1, R00.8, T82.1, Z45.00, Z45.01, Z95.0 |
| Chronic Pulmonary Disease | 3 | I27.8, I27.9, J40, J41, J42, J43, J44, J45, J46, J47, J60, J61, J62, J63, J64, J65, J66, J67, J68.4, J70.1, J70.3 |
| Chronic Renal Failure | 6 | I12.0, I31.1, N18, N19, N25.0, Z49.0, Z49.1, Z49.2, Z94.0, Z99.2 |
| Coagulopathy | 11 | D65, D66, D67, D68, D69.1, D69.3, D69.4, D69.5, D69.6 |
| Congestive Heart Failure | 9 | I09.0, I11.0, I13.0, I13.2, I25.5, I42.0, I42.1, I42.2, I42.5, I42.6, I42.7, I42.8, I42.9, I43, I50 |
| Deficiency Anemia | -2 | D50.8, D50.9, D51, D52, D53 |
| Depression | -5 | F20.4, F31.3 - F31.5, F32, F33, F34.1, F41.2, F43.2 |
| Diabetes Mellitus, Uncomplicated | 0 | E10.0, E10.1, E10.9, E11.0, E11.1, E11.9, E12.0, E12.1, E12.9, E13.0, E13.1, E13.9, E14.0, E14.1, E14.9 (excluding E10.2, E10.3, E10.4, E10.5, E10.6, E10.7, E10.8, E11.2, E11.3, E11.4, E11.5, E11.6, E11.7, E11.8, E12.2, E12.3, E12.4, E12.5, E12.6, E12.7, E12.8, E13.2, E13.3, E13.4, E13.5, E13.6, E13.7, E13.8, E14.2, E14.3, E14.4, E14.5, E14.6, E14.7, E14.8) |
| Diabetes Mellitus, Complicated | -3 | E10.2, E10.3, E10.4, E10.5, E10.6, E10.7, E10.8, E11.2, E11.3, E11.4, E11.5, E11.6, E11.7, E11.8, E12.2, E12.3, E12.4, E12.5, E12.6, E12.7, E12.8, E13.2, E13.3, E13.4, E13.5, E13.6, E13.7, E13.8, E14.2, E14.3, E14.4, E14.5, E14.6, E14.7, E14.8 |
| Drug Abuse | -7 | F11, F12, F13, F14, F15, F16, F18, F19, Z71.5, Z72.2 |
| Fluid And Electrolyte Disorders | 11 | E22.2, E86, E87 |
| Hypertension (combined uncomplicated and complicated) | -1 | I10, I11, I12, I13, I15 |
| Hypothyroidism | 0 | E00, E01, E02, E03, E89.0 |
| Liver Disease | 4 | B18, I85, I86.4, I98.2, K70, K71.1, K71.3, K71.4, K71.5, K71.7, K72, K73, K74, K76.0, K76.2, K76.9, Z94.4 |
| Lymphoma | 6 | C81, C82, C83, C84, C85, C88, C96, C90.0, C90.2 |
| Metastatic Cancer | 14 | C77, C78, C79, C80 |
| Neurological Disorders, other | 5 | G10, G11, G12, G13. G20, G21, G22, G25.4, G25.5, G31.2, G31.8, G31.9, G32, G35, G36, G37, G40, G41, G93.1, G93.4, R47.0, R56 |
| Obesity | -5 | E66 |
| Paralysis | 5 | G04.1, G11.4, G80.1, G80.2, G81, G82, G83.0, G83.1, G83.2, G83.3, G83.4, G83.9 |
| Peptic Ulcer Disease, Excluding Bleeding | 0 | K25.7, K25.9, K26.7, K26.9, K27.7, K27.9, K28.7, K28.9 |
| Peripheral Vascular Disorders | 3 | I70, I71, I73.1, I73.8, I73.9, I77.1, I79.0, I79.2, Z95.81, Z95.88, Z95.9 |
| Psychoses | -5 | F20, F22, F23, F24, F25, F28, F29, F30.2, F31.2, F31.5 |
| Pulmonary Circulation Disorder | 6 | I26, I27, I28.0, I28.8, I28.9 |
| Rheumatoid Arthritis / Collagen Vascular Diseases | 0 | L94.0, L94.1, L94.3, M05, M06, M08, M12.0, M12.3, M30, M31.0, M31.1, M31.2, M31.3, M32, M33, M34, M35, M45, M46.1, M46.8, M46.9 |
| Solid Tumor Without Metastases | 7 | C00, C01, C02, C03, C04, C05, C06, C07, C08, C09, C10, C11, C12, C13, C14, C15, C16, C17, C18, C19, C20, C21, C22, C23, C24, C25, C26, C30, C31, C32, C33, C34, C37, C38, C39, C40, C41, C43, C45, C46, C47, C48, C49, C50, C51, C52, C53, C54, C55, C56, C57, C58, C60, C61, C62, C63, C64, C65, C66, C67, C68, C69, C70, C71, C72, C73, C74, C75, C76, C97 |
| Valvular Heart Disease | 0 | I05, I06, I07, I08, I09.1, I34, I35, I36, I37, I38, I39, Q23.0, Q23.1, Q23.2, Q23.3, Z95.2, Z95.3, Z95.4 |
| Weight Loss | 9 | E40, E41, E42, E43, E44, E45, E46, R63.4, R64 |

* International Statistical Classification of Diseases and Related Health Problems (ICD-10-GM [German Modification])

Supplemental Table 2: Detailed baseline characteristics overall and comparing the first pandemic wave with the period thereafter

| **Proportion (*n*)** | | | |
| --- | --- | --- | --- |
| **Variable** | **First wave** | **After first wave** | ***P* value** |
| **Age** | | | |
| *mean (SD)* [years] | 68.5 ±17.2 | 64.4 ±22.6 | < 0.01 |
| ≤ 59 years | 28.3% (510) | 33.1% (23,355) | < 0.01 |
| 60−69 years | 16.1% (290) | 15.5% (10,943) | 0.14 |
| 70−79 years | 23.1% (417) | 19.5% (13,772) | < 0.01 |
| ≥ 80 years | 32.5% (586) | 32.0% (22,586) | 0.96 |
| **Sex** | | | |
| male | 51.9% (936) | 51.2% (36,190) |  |
| female | 48.1% (867) | 48.8% (34,466) | 0.35 |
| **SARI** | | | |
| no | 14.8% (267) | 46.7% (33,007) |  |
| yes | 85.2% (1,536) | 53.3% (37,649) | < 0.01 |
| **Elixhauser comorbidity index** | | | |
| *mean (SD)* | 11.0 ±11.6 | 9.8 ±11.2 | < 0.01 |
| < 0 | 13.3% (240) | 13.8% (9,724) | 0.78 |
| 0 | 16.7% (301) | 19.9% (14,066) | < 0.01 |
| 1-4 | 5.5% (99) | 5.5% (3,899) | 0.87 |
| ≥ 5 | 64.5% (1,163) | 60.8% (42,967) | < 0.01 |
| **Congestive heart failure** | | | |
| no | 77.4% (1,395) | 78.4% (55,403) |  |
| yes | 22.6% (408) | 21.6% (15,253) | < 0.01 |
| **Cardiac arrhythmias** | | | |
| no | 75.3% (1,357) | 75.6% (53,422) |  |
| yes | 24.7% (446) | 24.4% (17,234) | 0.34 |
| **Valvular disease** | | | |
| no | 94.5% (1,704) | 92.9% (65,621) |  |
| yes | 5.5% (99) | 7.1% (5,035) | 0.20 |
| **Pulmonary circulation disorders** | | | |
| no | 95.8% (1,727) | 95.2% (67,265) |  |
| yes | 4.2% (76) | 4.8% (3,391) | 0.86 |
| **Peripheral vascular disorders** | | | |
| no | 93.5% (1,685) | 92.5% (65,351) |  |
| yes | 6.5% (118) | 7.5% (5,305) | 0.43 |
| **Hypertension, uncomplicated** | | | |
| no | 56.0% (1,009) | 59.5% (42,013) |  |
| yes | 44.0% (794) | 40.5% (28,643) | < 0.01 |
| **Hypertension, complicated** | | | |
| no | 89.4% (1,612) | 88.4% (62,470) |  |
| yes | 10.6% (191) | 11.6% (8,186) | 0.07 |
| **Paralysis** | | | |
| no | 94.9% (1,711) | 95.4% (67,425) |  |
| yes | 5.1% (92) | 4.6% (3,231) | 0.22 |
| **Other neurological disorders** | | | |
| no | 92.1% (1,660) | 91.6% (64,756) |  |
| yes | 7.9% (143) | 8.4% (5,900) | 0.65 |
| **Chronic pulmonary disease** | | | |
| no | 88.4% (1,593) | 88.8% (62,764) |  |
| yes | 11.6% (210) | 11.2% (7,892) | 0.58 |
| **Diabetes, uncomplicated** | | | |
| no | 86.0% (1,550) | 84.2% (59,488) |  |
| yes | 14.0% (253) | 15.8% (11,168) | 0.60 |
| **Diabetes, complicated** | | | |
| no | 89.1% (1,607) | 90.1% (63,682) |  |
| yes | 10.9% (196) | 9.9% (6,974) | 0.09 |
| **Hypothyroidism** | | | |
| no | 85.4% (1,540) | 88.1% (62,220) |  |
| yes | 14.6% (263) | 11.9% (8,436) | < 0.01 |
| **Renal failure** | | | |
| no | 73.5% (1,325) | 73.0% (51,612) |  |
| yes | 26.5% (478) | 27.0% (19,044) | 0.04 |
| **Liver disease** | | | |
| no | 96.0% (1,731) | 96.1% (67,876) |  |
| yes | 4.0% (72) | 3.9% (2,780) | 0.73 |
| **Peptic ulcer disease excluding bleeding** | | | |
| no | 100.0% (1,803) | 99.9% (70,596) |  |
| yes | 0.0% (0) | 0.1% (60) | < 0.01 |
| **AIDS/HIV** | | | |
| no | 100.0% (1,803) | 100.0% (70,636) |  |
| yes | 0.0% (0) | 0.0% (20) | 0.16 |
| **Lymphoma** | | | |
| no | 99.3% (1,790) | 99.0% (69,969) |  |
| yes | 0.7% (13) | 1.0% (687) | 0.52 |
| **Metastatic cancer** | | | |
| no | 97.9% (1,766) | 97.6% (68,943) |  |
| yes | 2.1% (37) | 2.4% (1,713) | 0.79 |
| **Solid tumour without metastasis** | | | |
| no | 95.6% (1,724) | 94.7% (66,911) |  |
| yes | 4.4% (79) | 5.3% (3,745) | 0.60 |
| **Rheumatoid artritis/collaged vascular disease** | | | |
| no | 98.3% (1,772) | 98.1% (69,303) |  |
| yes | 1.7% (31) | 1.9% (1,353) | 0.43 |
| **Coagulopathy** | | | |
| no | 94.7% (1,707) | 95.3% (67,309) |  |
| yes | 5.3% (96) | 4.7% (3,347) | 0.07 |
| **Obesity** | | | |
| no | 88.6% (1,598) | 88.4% (62,453) |  |
| yes | 11.4% (205) | 11.6% (8,203) | 0.05 |
| **Weight loss** | | | |
| no | 86.6% (1,562) | 90.6% (64,015) |  |
| yes | 13.4% (241) | 9.4% (6,641) | < 0.01 |
| **Fluid and electrolyte disorders** | | | |
| no | 50.7% (915) | 60.0% (42,423) |  |
| yes | 49.3% (888) | 40.0% (28,233) | < 0.01 |
| **Blood loss anaemia** | | | |
| no | 99.3% (1,791) | 99.5% (70,305) |  |
| yes | 0.7% (12) | 0.5% (351) | 0.21 |
| **Deficiency anaemia** | | | |
| no | 96.3% (1,737) | 96.7% (68,296) |  |
| yes | 3.7% (66) | 3.3% (2,360) | 0.92 |
| **Alcohol abuse** | | | |
| no | 98.5% (1,776) | 97.7% (69,026) |  |
| yes | 1.5% (27) | 2.3% (1,630) | 0.08 |
| **Drug abuse** | | | |
| no | 99.6% (1,796) | 99.4% (70,221) |  |
| yes | 0.4% (7) | 0.6% (435) | 0.60 |
| **Psychoses** | | | |
| no | 98.2% (1,771) | 98.9% (69,872) |  |
| yes | 1.8% (32) | 1.1% (784) | < 0.01 |
| **Depression** | | | |
| no | 92.2% (1,662) | 94.0% (66,431) |  |
| yes | 7.8% (141) | 6.0% (4,225) | < 0.01 |

SARI: Severe acute respiratory infections

**Supplemental Table 3: Comparison of baseline characteristics dividing four pandemic periods based on incident new SARS-CoV-2 cases**

|  |  |  | |  | |  | |  | | |
| --- | --- | --- | --- | --- | --- | --- | --- | --- | --- | --- |
| **Variable** | | **First Wave**  **Proportion (*n*)** | **Wildtype after first wave**  **Proportion (*n*)** | ***P* value** | **Alpha**  **Proportion (*n*)** | ***P* value** | **Delta**  **Proportion (*n*)** | ***P* value** | **Omicron**  **Proportion (*n*)** | ***P* value** |
| **Age** | | | | | | | | | | |
| *Mean (SD)* | | 68.5 ±17.2 | 69.3 ±18.6 | 0.08 | 61.7 ±19.5 | < 0.01 | 62.7 ±22.1 | < 0.01 | 62.4 ±25.4 | < 0.01 |
| ≤ 59 years | | 28.3% (510) | 25.2% (5,196) | 0.03 | 41.2% (3,314) | < 0.01 | 37.6% (4,652) | < 0.01 | 34.4% (10,193) | < 0.01 |
| 60−69 years | | 16.1% (290) | 15.4% (3,168) | 0.11 | 19.3% (1,557) | 0.01 | 16.4% (2,031) | 0.55 | 14.1% (4,187) | < 0.01 |
| 70−79 years | | 23.1% (417) | 21.9% (4,509) | 0.11 | 19.7% (1,584) | < 0.01 | 17.9% (2,221) | < 0.01 | 18.4% (5,458) | < 0.01 |
| ≥ 80 years | | 32.5% (586) | 37.5% (7,733) | < 0.01 | 19.8% (1,593) | < 0.01 | 28.1% (3,474) | < 0.01 | 33.0% (9,786) | 0.25 |
| **Sex** | | | | | | | | | | |
| Male | | 51.9% (936) | 51.3% (10,568) |  | 54.0% (4,347) |  | 52.7% (6,519) |  | 49.8% (14,756) |  |
| Female | | 48.1% (867) | 48.7% (10,038) | 0.33 | 46.0% (3,701) | 0.19 | 47.3% (5,859) | 0.81 | 50.2% (14,868) | 0.05 |
| **SARI** | | | | | | | | | | |
| no | | 14.8% (267) | 30.1% (6,205) |  | 23.9% (1,926) |  | 34.2% (4,238) |  | 69.7% (20,638) |  |
| yes | | 85.2% (1,536) | 69.9% (14,401) | < 0.01 | 76.1% (6,122) | < 0.01 | 65.8% (8,140) | < 0.01 | 30.3% (8,986) | < 0.01 |
| **Elixhauser comorbidity index** | | | | | | | | | | |
| *Mean (SD)* | | 11.0 ±11.6 | 11.6 ±11.6 | 0.50 | 9.1 ±10.7 | < 0.01 | 9.7 ±11.1 | < 0.01 | 8.9 ±10.9 | < 0.01 |
| < 0 | | 13.3% (240) | 12.8% (2,628) | 0.38 | 15.3% (1,229) | 0.06 | 13.0% (1,607) | 0.51 | 14.4% (4,260) | 0.33 |
| 0 | | 16.7% (301) | 14.5% (2,978) | 0.18 | 19.8% (1,597) | < 0.01 | 21.3% (2,636) | < 0.01 | 23.1% (6,855) | < 0.01 |
| 1-4 | | 5.5% (99) | 5.3% (1,086) | 0.71 | 5.4% (435) | 0.92 | 4.9% (603) | 0.31 | 6.0% (1,775) | 0.31 |
| ≥ 5 | | 64.5% (1,163) | 67.5% (13,914) | 0.11 | 59.5% (4,787) | < 0.01 | 60.8% (7,532) | < 0.01 | 56.5% (16,734) | < 0.01 |
| **Congestive heart failure** | | | | | | | | | | |
| no | | 77.4% (1,395) | 73.9% (15,222) |  | 82.3% (6,623) |  | 80.3% (9,939) |  | 79.7% (23,619) |  |
| yes | | 22.6% (408) | 26.1% (5,384) | 0.22 | 17.7% (1,425) | < 0.01 | 19.7% (2,439) | < 0.01 | 20.3% (6,005) | < 0.01 |
| **Cardiac arrhythmias** | | | | | | | | | | |
| no | | 75.3% (1,357) | 72.0% (14,828) |  | 80.0% (6,436) |  | 77.2% (9,558) |  | 76.3% (22,600) |  |
| yes | | 24.7% (446) | 28.0% (5,778) | 0.04 | 20.0% (1,612) | < 0.01 | 22.8% (2,820) | < 0.01 | 23.7% (7,024) | 0.20 |
| **Valvular disease** | | | | | | | | | | |
| no | | 94.5% (1,704) | 91.3% (18,813) |  | 94.3% (7,593) |  | 93.9% (11,623) |  | 93.1% (27,592) |  |
| yes | | 5.5% (99) | 8.7% (1,793) | < 0.01 | 5.7% (455) | 0.23 | 6.1% (755) | 0.37 | 6.9% (2,032) | 0.21 |
| **Pulmonary circulation disorders** | | | | | | | | | | |
| no | | 95.8% (1,727) | 94.7% (19,508) |  | 94.7% (7,624) |  | 94.4% (11,683) |  | 96.0% (28,450) |  |
| yes | | 4.2% (76) | 5.3% (1,098) | 0.43 | 5.3% (424) | 0.30 | 5.6% (695) | 0.18 | 4.0% (1,174) | 0.25 |
| **Peripheral vascular disorders** | | | | | | | | | | |
| no | | 93.5% (1,685) | 91.5% (18,847) |  | 94.8% (7,629) |  | 93.4% (11,564) |  | 92.2% (27,311) |  |
| yes | | 6.5% (118) | 8.5% (1,759) | 0.06 | 5.2% (419) | < 0.01 | 6.6% (814) | 0.24 | 7.8% (2,313) | 0.13 |
| **Hypertension, uncomplicated** | | | | | | | | | | |
| no | | 56.0% (1,009) | 54.4% (11,216) |  | 59.2% (4,768) |  | 61.5% (7,617) |  | 62.2% (18,412) |  |
| yes | | 44.0% (794) | 45.6% (9,390) | 0.30 | 40.8% (3,280) | 0.02 | 38.5% (4,761) | < 0.01 | 37.8% (11,212) | < 0.01 |
| **Hypertension, complicated** | | | | | | | | | | |
| no | | 89.4% (1,612) | 86.7% (17,856) |  | 89.2% (7,175) |  | 89.6% (11,089) |  | 88.9% (26,350) |  |
| yes | | 10.6% (191) | 13.3% (2,750) | 0.56 | 10.8% (873) | < 0.01 | 10.4% (1,289) | < 0.01 | 11.1% (3,274) | 0.01 |
| **Paralysis** | | | | | | | | | | |
| no | | 94.9% (1,711) | 94.9% (19,545) |  | 96.6% (7,776) |  | 95.8% (11,852) |  | 95.4% (28,252) |  |
| yes | | 5.1% (92) | 5.1% (1,061) | 0.99 | 3.4% (272) | < 0.01 | 4.2% (526) | 0.02 | 4.6% (1,372) | 0.33 |
| **Other neurological disorders** | | | | | | | | | | |
| no | | 92.1% (1,660) | 90.7% (18,680) |  | 94.2% (7,579) |  | 93.1% (11,520) |  | 91.1% (26,977) |  |
| yes | | 7.9% (143) | 9.3% (1,926) | 0.07 | 5.8% (469) | < 0.01 | 6.9% (858) | 0.05 | 8.9% (2,647) | 0.20 |
| **Chronic pulmonary disease** | | | | | | | | | | |
| no | | 88.4% (1,593) | 87.7% (18,067) |  | 89.3% (7,189) |  | 89.6% (11,087) |  | 89.2% (26,421) |  |
| yes | | 11.6% (210) | 12.3% (2,539) | 0.42 | 10.7% (859) | 0.25 | 10.4% (1,291) | 0.14 | 10.8% (3,203) | 0.31 |
| **Diabetes, uncomplicated** | | | | | | | | | | |
| no | | 86.0% (1,550) | 82.2% (16,937) |  | 82.6% (6,644) |  | 83.4% (10,321) |  | 86.4% (25,586) |  |
| yes | | 14.0% (253) | 17.8% (3,669) | 0.04 | 17.4% (1,404) | 0.04 | 16.6% (2,057) | 0.44 | 13.6% (4,038) | 0.16 |
| **Diabetes, complicated** | | | | | | | | | | |
| no | | 89.1% (1,607) | 87.2% (17,965) |  | 91.2% (7,342) |  | 91.5% (11,328) |  | 91.3% (27,047) |  |
| yes | | 10.9% (196) | 12.8% (2,641) | 0.03 | 8.8% (706) | < 0.01 | 8.5% (1,050) | < 0.01 | 8.7% (2,577) | < 0.01 |
| **Hypothyroidism** | | | | | | | | | | |
| no | | 85.4% (1,540) | 87.0% (17,924) |  | 88.2% (7,099) |  | 88.6% (10,969) |  | 88.5% (26,228) |  |
| yes | | 14.6% (263) | 13.0% (2,682) | 0.19 | 11.8% (949) | < 0.01 | 11.4% (1,409) | < 0.01 | 11.5% (3,396) | < 0.01 |
| **Renal failure** | | | | | | | | | | |
| no | | 73.5% (1,325) | 66.5% (13,710) |  | 78.2% (6,290) |  | 75.7% (9,370) |  | 75.1% (22,242) |  |
| yes | | 26.5% (478) | 33.5% (6,896) | < 0.01 | 21.8% (1,758) | < 0.01 | 24.3% (3,008) | < 0.01 | 24.9% (7,382) | < 0.01 |
| **Liver disease** | | | | | | | | | | |
| no | | 96.0% (1,731) | 95.6% (19,700) |  | 96.0% (7,726) |  | 96.0% (11,885) |  | 96.4% (28,565) |  |
| yes | | 4.0% (72) | 4.4% (906) | 0.61 | 4.0% (322) | 0.81 | 4.0% (493) | 0.64 | 3.6% (1,059) | 0.33 |
| **Peptic ulcer disease excluding bleeding** | | | | | | | | | | |
| no | | 100.0% (1,803) | 99.9% (20,586) |  | 100.0% (8,044) |  | 99.9% (12,368) |  | 99.9% (29,598) |  |
| yes | | 0.0% (0) | 0.1% (20) | < 0.01 | 0.0% (4) | < 0.01 | 0.1% (10) | < 0.01 | 0.1% (26) | < 0.01 |
| **AIDS/HIV** | | | | | | | | | | |
| no | | 100.0% (1,803) | 100.0% (20,599) |  | 100.0% (8,045) |  | 100.0% (12,378) |  | 100.0% (29,614) |  |
| yes | | 0.0% (0) | 0.0% (7) | 0.01 | 0.0% (3) | 0.01 | 0.0% (0) | 1.00 | 0.0% (10) | 0.01 |
| **Lymphoma** | | | | | | | | | | |
| no | | 99.3% (1,790) | 99.2% (20,447) |  | 99.3% (7,990) |  | 99.1% (12,262) |  | 98.8% (29,270) |  |
| yes | | 0.7% (13) | 0.8% (159) | 0.70 | 0.7% (58) | 0.78 | 0.9% (116) | 0.69 | 1.2% (354) | 0.13 |
| **Metastatic cancer** | | | | | | | | | | |
| no | | 97.9% (1,766) | 97.4% (20,069) |  | 98.6% (7,938) |  | 98.0% (12,134) |  | 97.2% (28,802) |  |
| yes | | 2.1% (37) | 2.6% (537) | 0.61 | 1.4% (110) | < 0.01 | 2.0% (244) | 0.27 | 2.8% (822) | 0.22 |
| **Solid tumour without metastasis** | | | | | | | | | | |
| no | | 95.6% (1,724) | 94.5% (19,472) |  | 96.6% (7,777) |  | 95.6% (11,829) |  | 94.0% (27,833) |  |
| yes | | 4.4% (79) | 5.5% (1,134) | 0.57 | 3.4% (271) | < 0.01 | 4.4% (549) | 0.22 | 6.0% (1,791) | 0.06 |
| **Rheumatoid artritis/collaged vascular disease** | | | | | | | | | | |
| no | | 98.3% (1,772) | 98.1% (20,220) |  | 98.3% (7,910) |  | 98.1% (12,148) |  | 98.0% (29,025) |  |
| yes | | 1.7% (31) | 1.9% (386) | 0.54 | 1.7% (138) | 0.74 | 1.9% (230) | 0.57 | 2.0% (599) | 0.26 |
| **Coagulopathy** | | | | | | | | | | |
| no | | 94.7% (1,707) | 94.5% (19,480) |  | 94.2% (7,581) |  | 94.7% (11,716) |  | 96.3% (28,532) |  |
| yes | | 5.3% (96) | 5.5% (1,126) | 0.64 | 5.8% (467) | 0.65 | 5.3% (662) | 0.65 | 3.7% (1,092) | < 0.01 |
| **Obesity** | | | | | | | | | | |
| no | | 88.6% (1,598) | 87.3% (17,987) |  | 83.6% (6,727) |  | 87.0% (10,767) |  | 91.0% (26,972) |  |
| yes | | 11.4% (205) | 12.7% (2,619) | 0.55 | 16.4% (1,321) | < 0.01 | 13.0% (1,611) | 0.44 | 9.0% (2,652) | < 0.01 |
| **Weight loss** | | | | | | | | | | |
| no | | 86.6% (1,562) | 87.9% (18,114) |  | 90.7% (7,300) |  | 90.7% (11,230) |  | 92.4% (27,371) |  |
| yes | | 13.4% (241) | 12.1% (2,492) | < 0.01 | 9.3% (748) | < 0.01 | 9.3% (1,148) | < 0.01 | 7.6% (2,253) | < 0.01 |
| **Fluid and electrolyte disorders** | | | | | | | | | | |
| no | | 50.7% (915) | 54.1% (11,138) |  | 56.3% (4,535) |  | 57.1% (7,071) |  | 66.4% (19,679) |  |
| yes | | 49.3% (888) | 45.9% (9,468) | 0.10 | 43.7% (3,513) | < 0.01 | 42.9% (5,307) | < 0.01 | 33.6% (9,945) | < 0.01 |
| **Blood loss anaemia** | | | | | | | | | | |
| no | | 99.3% (1,791) | 99.4% (20,487) |  | 99.7% (8,025) |  | 99.6% (12,331) |  | 99.5% (29,462) |  |
| yes | | 0.7% (12) | 0.6% (119) | 0.51 | 0.3% (23) | 0.01 | 0.4% (47) | 0.07 | 0.5% (162) | 0.32 |
| **Deficiency anaemia** | | | | | | | | | | |
| no | | 96.3% (1,737) | 96.2% (19,829) |  | 97.4% (7,836) |  | 97.3% (12,046) |  | 96.5% (28,585) |  |
| yes | | 3.7% (66) | 3.8% (777) | 0.54 | 2.6% (212) | 0.08 | 2.7% (332) | 0.11 | 3.5% (1,039) | 0.76 |
| **Alcohol abuse** | | | | | | | | | | |
| no | | 98.5% (1,776) | 97.9% (20,174) |  | 98.5% (7,925) |  | 97.8% (12,101) |  | 97.3% (28,826) |  |
| yes | | 1.5% (27) | 2.1% (432) | 0.27 | 1.5% (123) | 0.69 | 2.2% (277) | 0.15 | 2.7% (798) | < 0.01 |
| **Drug abuse** | | | | | | | | | | |
| no | | 99.6% (1,796) | 99.6% (20,522) |  | 99.7% (8,024) |  | 99.5% (12,322) |  | 99.1% (29,353) |  |
| yes | | 0.4% (7) | 0.4% (84) | 0.56 | 0.3% (24) | 0.23 | 0.5% (56) | 0.87 | 0.9% (271) | 0.10 |
| **Psychoses** | | | | | | | | | | |
| no | | 98.2% (1,771) | 98.8% (20,355) |  | 99.3% (7,992) |  | 98.9% (12,246) |  | 98.8% (29,279) |  |
| yes | | 1.8% (32) | 1.2% (251) | 0.02 | 0.7% (56) | < 0.01 | 1.1% (132) | < 0.01 | 1.2% (345) | < 0.01 |
| **Depression** | | | | | | | | | | |
| no | | 92.2% (1,662) | 93.4% (19,237) |  | 94.8% (7,633) |  | 93.9% (11,625) |  | 94.3% (27,936) |  |
| yes | | 7.8% (141) | 6.6% (1,369) | 0.07 | 5.2% (415) | < 0.01 | 6.1% (753) | < 0.01 | 5.7% (1,688) | < 0.01 |

P values behind wave columns refer to a comparison of the corresponding pandemic period with the first waves’ data

**Supplemental Table 4: Comparison of in-hospital outcomes dividing four pandemic periods based on incident new SARS-CoV-2 cases**

| **Variable** | **First Wave**  **Proportion (*n*)** | **Wildtype after first wave**  **Proportion (*n*)** | ***P* value** | **Alpha**  **Proportion (*n*)** | ***P* value** | **Delta**  **Proportion (*n*)** | ***P* value** | **Omicron**  **Proportion (*n*)** | ***P* value** |
| --- | --- | --- | --- | --- | --- | --- | --- | --- | --- |
| **Intensive care** | | | | | | | | | |
| yes | 33.1% (597) | 26.3% (5,413) | <0.01 | 27.3% (2,200) | <0.01 | 25.2% (3,125) | <0.01 | 16.2% (4,809) | <0.01 |
| **Mechanical ventilation** | | | | | | | | | |
| yes | 20.1% (3,460) | 16.8% (3,460) | <0.01 | 21.5% (1,733) | 0.60 | 18.7% (2,319) | 0.01 | 5.8% (1,715) | <0.01 |
| **ECMO** | | | | | | | | | |
| yes | 0.7% (13) | 0.5% (107) | <0.01 | 1.2% (94) | 0.78 | 1.0% (127) | 0.74 | 0.1% (37) | <0.01 |
| **In-hospital mortality*** | | | | | | | | | |
| yes | 22.4% (352) | 21.3% (4,061) | <0.01 | 14.7% (1,087) | <0.01 | 17.2% (1,990) | <0.01 | 7.7% (2,187) | <0.01 |

All Odds ratios and P values refer to a comparison of the corresponding pandemic period with the first waves’ data.

* Based on 67,913 cases after the exclusion of cases with hospital discharge type of hospital transfer or unspecified reason.

ECMO: extra-corporal membrane oxygenation

**Supplemental Table 5: Comparison of inpatient pathways and costs dividing four pandemic periods based on incident new SARS-CoV-2 cases**

| **Cohort** | **Mean (SD)** | **Median (IQR)** | **Ratio (95% CI)** | ***P* value** |
| --- | --- | --- | --- | --- |
| **Length of stay** | | | | |
| First wave | 14.6 ±26.4 | 10.0 [5, 18] |  |  |
| Wildtype after first wave | 13.0 ±14.8 | 8.0 [4, 16] | -0.13 (-0.17−-0.09) | < 0.01 |
| Alpha | 10.7 ±11.9 | 7.0 [4, 14] | -0.23 (-0.28−-0.19) | < 0.01 |
| Delta | 12.3 ±16.6 | 7.0 [3, 15] | -0.23 (-0.27−-0.18) | < 0.01 |
| Omicron | 9.3 ±12.1 | 5.0 [2, 11] | -0.46 (-0.50−-0.42) | < 0.01 |
| **Length of stay at ICU^*^** | | | | |
| First wave | 12.1 ±15.0 | 6.0 [2, 16] |  |  |
| Wildtype after first wave | 9.9 ±13.3 | 5.0 [2, 13] | -0.17 (-0.27−-0.08) | < 0.01 |
| Alpha | 11.0 ±12.6 | 7.0 [3, 14] | 0.01 (-0.09− 0.11) | 0.89 |
| Delta | 10.4 ±14.0 | 6.0 [2, 13] | -0.09 (-0.18− 0.01) | 0.09 |
| Omicron | 5.8 ±9.2 | 3.0 [1, 6] | -0.70 (-0.80−-0.61) | < 0.01 |
| **Duration of mechanical ventilation^†^** | | | | |
| First wave | 321.5 ±344.1 | 234.0 [68, 448] |  |  |
| Wildtype after first wave | 251.1 ±291.5 | 149.0 [49, 353] | -0.38 (-0.55−-0.20) | < 0.01 |
| Alpha | 262.8 ±298.9 | 159.0 [56, 364] | -0.26 (-0.45−-0.08) | < 0.01 |
| Delta | 249.6 ±304.9 | 149.0 [48, 332] | -0.37 (-0.55−-0.19) | < 0.01 |
| Omicron | 166.2 ±264.0 | 60.0 [15, 197] | -1.11 (-1.30−-0.93) | < 0.01 |
| **Costs of hospitalization^‡^** | | | | |
| First wave | 11,871.0 ±21,861.3 | 4,453.3 [2,983, 10,711] |  |  |
| Wildtype after first wave | 10,045.7 ±17,885.5 | 4,613.2 [3,112, 9,666] | -0.09 (-0.13−-0.05) | < 0.01 |
| Alpha | 9,768.0 ±16,695.2 | 4,017.1 [3,132, 8,348] | -0.10 (-0.14−-0.05) | < 0.01 |
| Delta | 10,150.5 ±19,539.3 | 4,039.2 [2,993, 9,003] | -0.12 (-0.16−-0.07) | < 0.01 |
| Omicron | 6,393.7 ±10,106.1 | 3,374.9 [2,333, 6,810] | -0.42 (-0.46−-0.37) | < 0.01 |

^*^ Based on 16,093 cases (22.2%). We excluded cases with length of stay at ICU = 0.

^†^ Based on 8,078 cases (11.1%). We excluded cases with duration of ventilation = 0.

^‡^ Based on 72,459 cases (100%). We excluded cases with costs = 0.

**Supplemental Figure 1: Graphical overview on pandemic development over time in Germany including public health interventions**


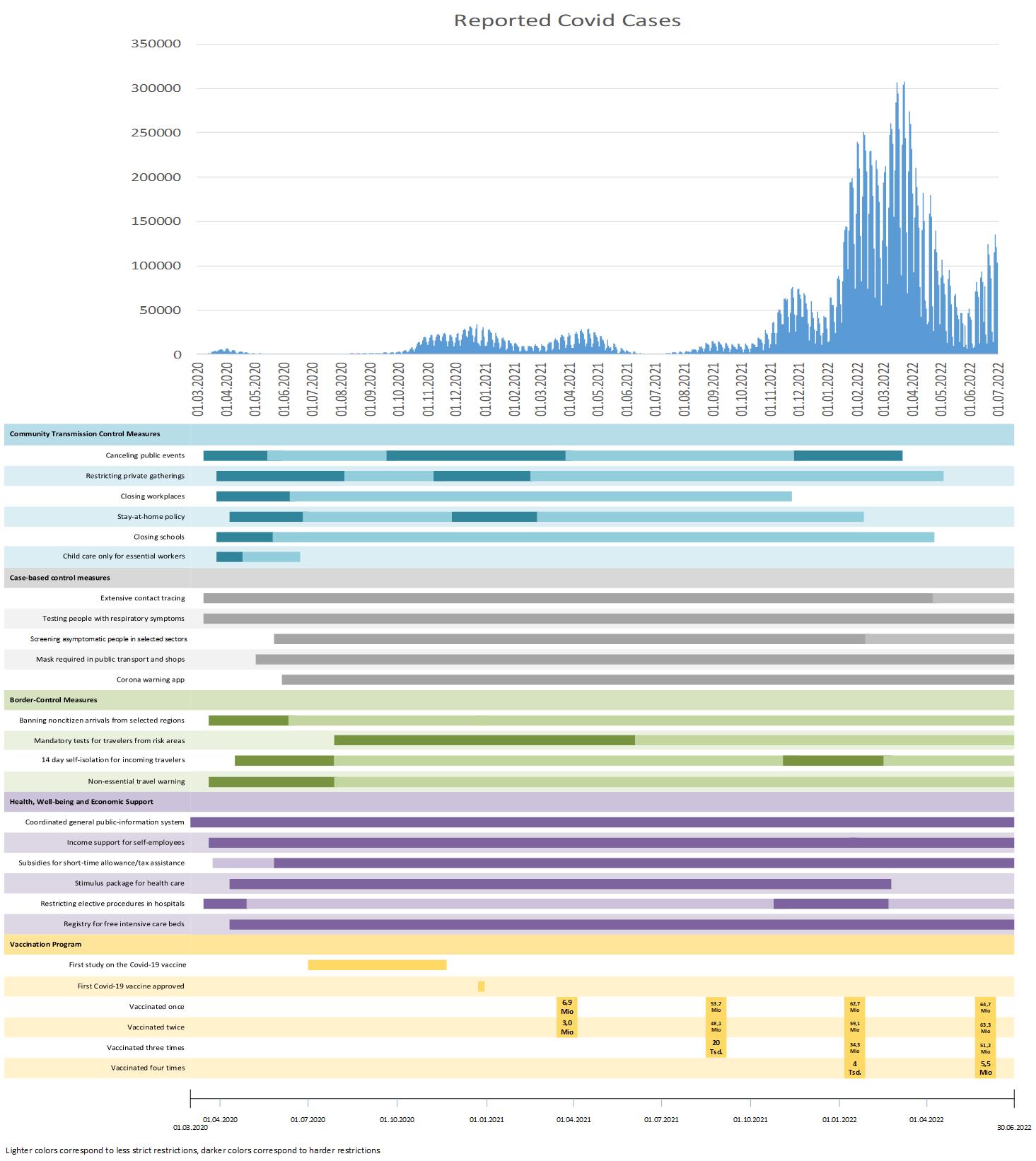


**Supplemental Figure 2: Sliding comparison of in-hospital mortality in 9-week periods with the first pandemic wave stratified for intensive care therapy**


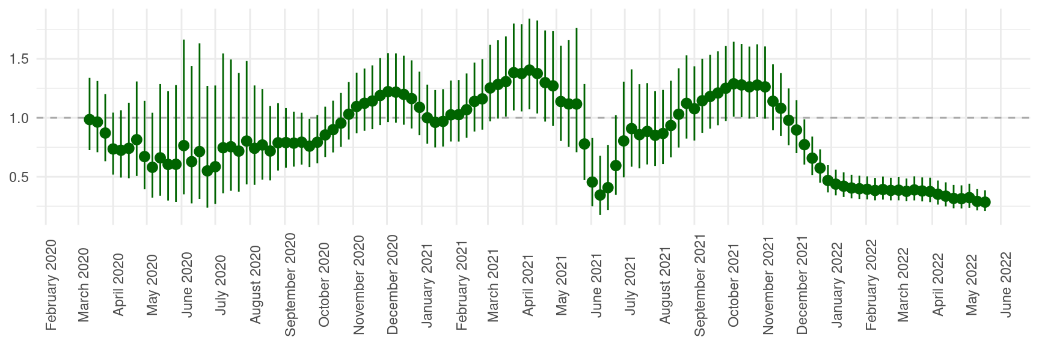

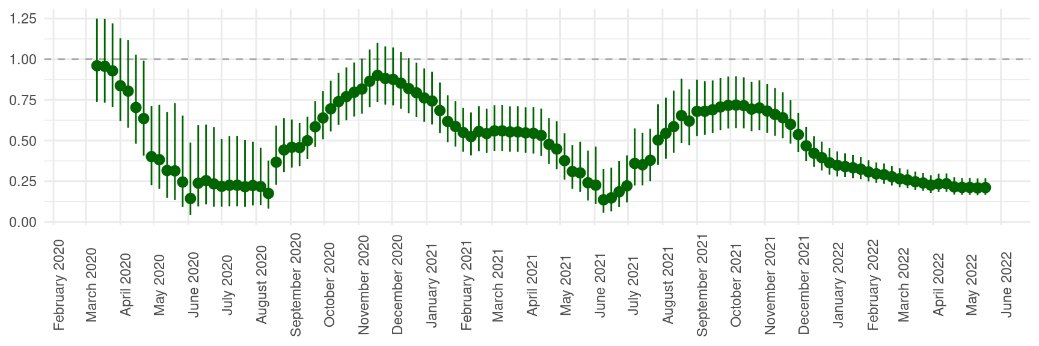


Sliding comparison of in-hospital mortality rates (adjusted for age, gender and Elixhauser comorbidity index) for patients receiving intensive care therapy (left panel) and those who did not (right panel)
